# Successful Esophago-Enterostomy

**Published:** 1898-02

**Authors:** 


### SUCCESSFUL ESOPHAGO-ENTEROSTOMY.

The late complete removal of the human stomach by Dr. Schlatter of Zurich, Switzerland, has been well exploited in the daily press. The *Medical Record* of December 25th contains a full account of the operation, as well as comments by Dr. Wendt of New York, who in a recent visit to Zurich met both operator and patient.

Hitherto operations on the stomach for the removal of malignant growths have taken away only portions of that organ, enough being allowed to remain to serve as a receptacle for food and for gastric digestion. In the present instance this was probably all that was at first anticipated, but when the abdomen was opened and the stomach found "diseased *in toto*," the operator decided to "attempt to excise the entire organ." The stomach was then severed at the esophageal and pyloric extremities. The free end of the duodenum was then invaginated and the opening closed by double suture. Then a knuckle of intestine, about fifteen inches from the beginning of the duodenum, was drawn up over the transverse colon and stitched to the extremity of the esophagus. The patient, who was a woman fifty-six years of age, bore the operation well, and steadily increased in weight after the removal of the cancerous stomach.

Advantage was taken of this case, in the course of her recovery, to make certain interesting physiological and clinical observations, and while broad generalizations cannot of course be made upon a single case, it seems justifiable (says Dr. Wendt) to formulate the following conclusions:

"The human stomach is not a vital organ.

"The digestive capacity of the human stomach has been considerably overrated.

"The fluids and solids constituting an ordinary mixed diet are capable of complete digestion and assimilation without the aid of the human stomach.

"A gain in the weight of the body may take place in spite of the total absence of gastric activity.

"Typical vomiting may occur without a stomach.

"The general health of a person need not immediately deteriorate on account of removal of the stomach.

"The most important office of the human stomach is to act as a reservoir for the reception, preliminary preparation, and propulsion of food and fluids. It also fulfils a useful purpose in regulating the temperature of swallowed solids and liquids.

"The chemical functions of the human stomach may be completely and satisfactorily performed by the other divisions of the alimentary canal.

"Gastric juice is hostile to the development of many micro-organisms.

"The free acid of normal gastric secretions has no power to arrest putrefactive changes in the intestinal track. Its antiseptic and bactericide potency has been overestimated."

